# Abstracts

**Published:** 1893-03

**Authors:** 


### Abstracts.

### SYMPTOMS OF SULFONAL POISONING.

DR. LÉPINE, of Lyons, France, in *La Semaine Medicale.* The symptoms are the same in all the cases. Prominent among them are: Gastro-intestinal disturbances, vomiting, and constipation; in some cases symptoms referable to the nervous system, such as failure of coördination and diminution of the reflexes; an exanthematous eruption in somewhat rare cases; lastly, diminished secretion of the urine, which is of a peculiar red color, and contains some biliary pigment, mucin, and albumin, with leucocytes, numerous red blood cells, and epithelial casts, a sign of renal inflammation. In one case, the urine examined by Jolles was acid, with a low percentage of chlorides and phosphates, but contained a large quantity of uric acid and indican; moreover, tyrosine was present. In exceptional cases, the breath smells of acetone. Death is usually due to syncope.

It is noteworthy that in all the cases of death from sulfonal poisoning which have been recorded, the patients were of the female sex.

In the absence of any marked pathological changes in the human subject, Dr. Kast has undertaken some experiments on animals.

In a first series of experiments performed on three dogs and three rabbits, sulfonal was given (in doses of from half a gramme to a gramme—eight to fifteen grains—in the case of the dogs,) until death occurred. This took place in about twenty days. The urine of the animals contained red blood corpuscles, but no hemato porphyrin. In a second series, the animals were killed early in the experiment, and in a third, poisoning was gradually induced in order to reproduce as closely as possible the condition found in man. In all these cases the kidneys were carefully examined after death, but no important change was discovered, the epithelium of the glomeruli and convoluted tubules was normal, but in dogs which had died of advanced poisoning, hemorrhage was found in the glomeruli, and the epithelium of some of the tubules was the seat of calcareous infiltration.

Dr. Kast's researches have shown that sulfonal becomes decomposed to a large extent in the body, and Dr. Smith has demonstrated that *sulphonic acid* was one of the products of this decomposition. What was wanted, therefore, was to ascertain whether this acid possessed any special toxic properties or not. For this purpose Dr. Kast administered on one occasion thirteen kilogrammes and four grammes (over twenty-six pounds avoirdupois) of sulphonic acid to a dog, and ten grammes (two and one-half drachms) on the following day. The urine was rendered very acid, but contained no pigment. The presence of hematoporphyrin is not, therefore, due to this acid.

Whatever may be the truth, a fact is now definitely established, viz., the danger which the patient runs whenever hematoporphyrin appears in the urine. As soon as this is detected, the administration of sulfonal should at once be stopped.

---

### CONTRIBUTIONS TO CEREBRAL SURGERY.

Drs. McBurney and Starr, of New York, made a joint report of three operations for cerebral neoplasms. Though unsuccessful, the authors felt that all such cases should be added to the literature in order that a true estimate of the value of cerebral surgery might ultimately be made.

The first case was one in which a diagnosis of a probable gumma of the left frontal lobe was made. The chief symptoms were a progressive development, within two years, of headache and nausea at times, localized pain, increasing hebetude, dimness of vision, with optic neuritis and exaggerated knee-jerk and ankle clonus upon the right side, with right hemiparesis. The operation was performed, and an encapsuled sarcoma was removed from the left frontal lobe, three and one-half by one and three-quarter inches in size. The patient died in eight hours. In connection with this case Dr. Starr took occasion to lay considerable stress upon mental symptoms as of value in the localization of frontal lobe lesions. Chief among the mental symptoms are a loss of self-control, lack of power of concentration, and psychical dulness. Such symptoms were present in a large number of cases collected by him.

The second case was one presenting the following symptoms: Choked disc, headache in the occipital and frontal regions, tinnitus aurium, vertigo, numbness of the left side of the face, drowsiness, nystagmus, diplopia, and mental dulness. Later there was optic atrophy, deafness in the left ear, staggering gait, weakness of the right hand, and staggering to the right in walking. On the right side the knee-jerk was exaggerated, and there was ankle clonus. As the symptoms pointed particularly to the cerebellum, an operation was performed, but nothing abnormal was found about the cerebellum, except evidence of great brain pressure. The wound was closed and remained aseptic, the patient recovering nicely from the effects of the operation. He died in about two weeks. At the autopsy a glio-sarcoma was found pressing upon the left half of the cerebellum and left half of the pons varolii.

The third case was one presenting the common symptoms of cerebellar tumor. The case was a child of seven. There was headache, vomiting, staggering gait, and optic neuritis. Upon operating, the exterior of the cerebellum was found to be normal. An aspirating needle introduced into its substance withdrew two drachms of clear fluid from a cyst. The child died suddenly six days after the operation. At the autopsy a small glio-sarcoma was discovered in the cerebellum and in its center the small cyst which had been evacuated.

One of the chief features in all of Dr. McBurney's operations upon the head is his method of opening the skull. He leaves the periosteum upon the bone and with the chisel cuts out such an area of bone as is required in his judgment, leaving the bone, however, attached by an isthmus which is broken through as he elevates the piece. Thus the piece of bone remains attached to the periosteum, and upon being replaced after the operation grows fast again without difficulty and with no interference with its nourishment. Besides the evident advantages of the procedure, it really takes less time than the operation with the trephine and bone-forceps.—*The Medical Week.*

---

### BREAD AND DYSPEPSIA.

The conclusion that wheat bread is unfit for dyspeptics, sometimes jumped at because ill effects are noticed to follow its use, is erroneous. On the contrary, it has been pointed out by Bouchard and others, that farinaceous food is peculiarly adapted to some dyspeptic patients. It is the microbes in the starch, which are capable of producing irritating acids, that cause the trouble. To avoid this, Bouchard recommends that only the crust or toasted crumb of the bread be used by dyspeptics, particularly those whose stomachs are dilated. The reason of this is explained by the fact that baking temporarily, though not permanently, arrests the fermentation of dough. When it is again heated by the warmth of the stomach, the fermentation is renewed. In cases where the bread is toasted brown through, the fermentation is stopped permanently.—*Food,* February, 1893.

